# The Underlying Changes in Serum Metabolic Profiles and Efficacy Prediction in Patients with Extensive Ulcerative Colitis Undergoing Fecal Microbiota Transplantation

**DOI:** 10.3390/nu15153340

**Published:** 2023-07-27

**Authors:** Xia Wu, Pan Li, Weihong Wang, Jie Xu, Rujun Ai, Quan Wen, Bota Cui, Faming Zhang

**Affiliations:** 1 Department of Microbiota Medicine, Medical Center for Digestive Diseases, The Second Affiliated Hospital of Nanjing Medical University, Nanjing 210011, China; wuxianjmu@163.com (X.W.); panli@njmu.edu.cn (P.L.); wwhome0703@163.com (W.W.); xujie1368@163.com (J.X.); airujun98@163.com (R.A.); wenquan@njmu.edu.cn (Q.W.); cuibota@njmu.edu.cn (B.C.); 2 Key Lab of Holistic Integrative Enterology, Nanjing Medical University, Nanjing 210011, China

**Keywords:** washed microbiota transplantation, inflammatory bowel disease, extensive UC, refractory ulcerative colitis, serum metabolomics, machine learning

## Abstract

(1) Background: Fecal microbiota transplantation (FMT) is an effective treatment for ulcerative colitis (UC). Metabolomic techniques would assist physicians in clinical decision-making. (2) Methods: Patients with active UC undergoing FMT were enrolled in the study and monitored for 3 months. We explored short-term changes in the serum metabolic signatures of groups and the association between baseline serum metabolomic profiles and patient outcomes. (3) Results: Forty-four eligible patients were included in the analysis. Of them, 50.0% and 29.5% achieved clinical response and clinical remission, respectively, 3 months post-FMT. The top two significantly altered pathways in the response group were vitamin B6 metabolism and aminoacyl-tRNA biosynthesis. Both the remission and response groups exhibited an altered and enriched pathway for the biosynthesis of primary bile acid. We found a clear separation between the remission and non-remission groups at baseline, characterized by the higher levels of glycerophosphocholines, glycerophospholipids, and glycerophosphoethanolamines in the remission group. A random forest (RF) classifier was constructed with 20 metabolic markers selected by the Boruta method to predict clinical remission 3 months post-FMT, with an area under the curve of 0.963. (4) Conclusions: FMT effectively induced a response in patients with active UC, with metabolites partially improving post-FMT in the responsive group. A promising role of serum metabolites in the non-invasive prediction of FMT efficacy for UC demonstrated the value of metabolome-informed FMT in managing UC.

## 1. Introduction

Ulcerative colitis (UC) is a chronic inflammatory bowel disease (IBD) that involves relapsing and remitting symptoms, including bloody stools, diarrhea, and abdominal pain. The pathogenesis of UC is not fully understood, but it is widely believed to be related to the chronic disruption of gut homeostasis and immune disorders [1]. The crucial role of gut microbiota in UC makes microbial manipulation an attractive treatment for UC [1]. Increasing evidence from randomized controlled trials has suggested that fecal microbiota transplantation (FMT) is an effective and safe treatment for active UC [2,3,4] in the short term. We previously demonstrated that a second course of FMT within 4 months after the initial FMT would be beneficial for patients with UC to maintain long-term efficacy [5]. However, the underlying mechanisms and mediators of FMT remain unclear.

Although the use of FMT to treat IBD has received widespread attention, heterogeneous clinical outcomes necessitate a personalized treatment strategy to optimize efficacy and reduce costs. The distinct changes in gut microbiota profiles post-FMT observed in different patients with UC highlighted the possibility of using patients’ specific microbial and metabolomic biomarkers to promote successful therapeutic outcomes [6,7]. Metabolomic techniques provide insights into disease pathogenesis and therapeutic response assessment by elucidating disease-associated metabolic perturbations and changes in host metabolism due to therapeutic intervention [8]. These techniques allow indirect estimates of the functional influence of gut microbiota manipulation in hosts with IBD by measuring microbial metabolites or host-microbial co-metabolites [8]. In addition, the gut microbiota can also influence an individual’s response to drugs and food transformation by enzymatically transforming their structure or altering their bioavailability, bioactivity, or toxicity [9]. For example, the gut microbiota can indirectly impact an individual’s response to immunotherapy for the treatment of cancer [10]. The reconstruction of gut microbiota by FMT in UC patients would be expected to change their metabolic function, thus directly or indirectly affecting the individual-specific efficacy of FMT. Therefore, applying metabolomic techniques to produce a timely efficacy prediction for UC patients undergoing FMT would assist physicians in clinical decision-making.

Here, untargeted metabolomic analysis by liquid chromatography–mass spectrometry (LC–MS) was used to broadly assess metabolic changes and identify potential biomarkers in patients with UC treated by FMT. Our analysis will provide a reference for quantitative measurement of the identified biomarkers and then timely monitoring of the efficacy of FMT in the future, thereby laying the foundation for the establishment of a high-performance artificial intelligence evaluation model. We chose serum samples from our biobank due to their advantages of minimal sample preparation, having well-characterized biological profiles, lower susceptibility to dietary influence, and reflecting on systemic metabolism [8,11].

## 2. Materials and Methods

### 2.1. Study Design and Setting

**Figure 1:**
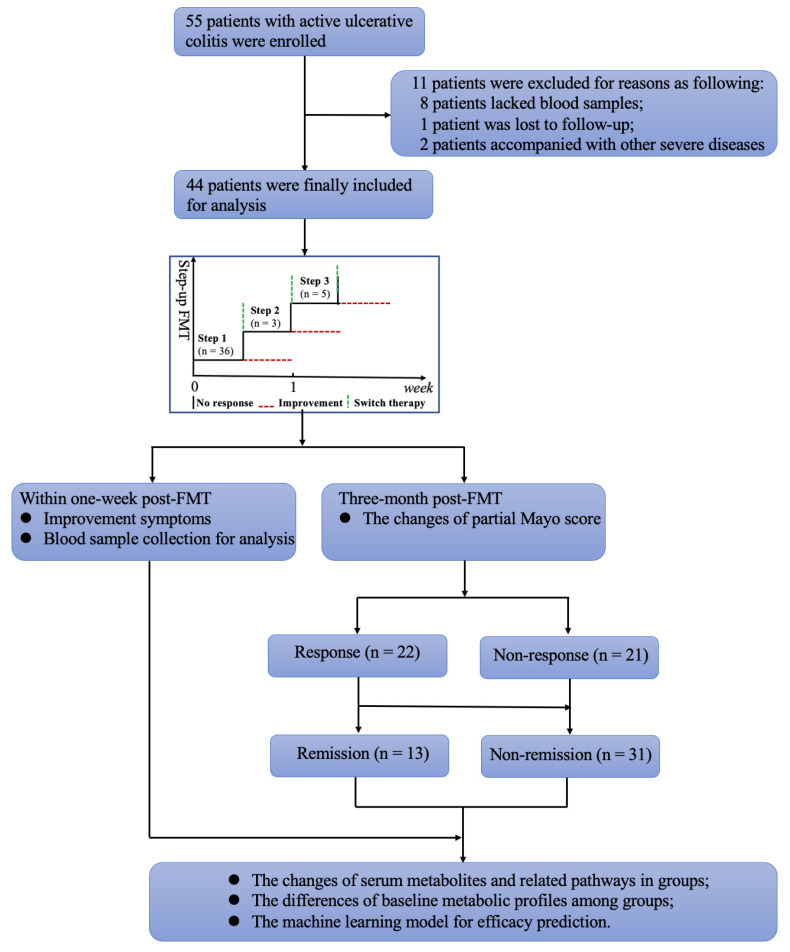
Flow chart of the study design. FMT, fecal microbiota transplantation.

This study was part of a registered trial (NCT01790061) and was conducted at the Second Affiliated Hospital of Nanjing Medical University. It was a retrospective analysis of a prospective trial. We selected the study cohort based on the availability of blood samples and associated data between May 2013 and April 2015. As shown in the flow chart in Figure 1, the patients’ demographics and clinical characteristics were thoroughly assessed before FMT. The daily score of abdominal pain and frequency of defecation were recorded at baseline and within 1 week post-FMT. The clinical efficacy of treatments was evaluated both 1 week and 3 months after the initial FMT.

### 2.2. Patients and Donors

The inclusion criteria for including patients in the study are the following: (1) Patients were diagnosed with UC based on typical clinical, endoscopic, and histological criteria; (2) patients either had active UC with a Mayo Score ≥ 3 and exhibited a poor response to conventional medications (e.g., 5-aminosalicylic acid (5-ASA), steroids, cyclosporine A, or biologics) or had acute severe UC defined as a Mayo Score ≥ 11; and (3) patients provided informed consent and underwent at least one course of FMT. Participants were excluded if any one of the following criteria (established before the study began) was satisfied: (1) patients missed blood sample collection; (2) patients had a follow-up period < 3 months; or (3) patients suffered from other intestinal diseases, e.g., *Clostridioides difficile* infection, or from other severe diseases, such as malignant neoplasm, serious liver or kidney disease, or cardiopulmonary failure.

To screen healthy donors for FMT, we employed methods described in our previous study [12]. All donors were from our hospital’s universal stool bank (Chinese fmtBank). Their ages ranged from 5 to 24 from 2013 to 2015.

### 2.3. FMT Procedure and Strategy

Prior to April 2014, we prepared fecal microbiota by manual filtration and centrifugation. Beginning in April 2014, we adopted a new methodology for FMT in our center as washed microbiota transplantation [13], which includes repeated processes of microfiltration, centrifugation, washing, discarding, and resuspension using an automatic purification system (GenFMTer; FMT Medical, Nanjing, China) and a Good Manufacturing Practice (GMP)-level laboratory room. The one-hour protocol we adopted limits the time from fecal donation to infusion of the fresh or frozen microbiota into the patient’s intestine within one hour. Five units of enriched microbiota precipitate were dissolved in 100 mL of normal saline [13,14] and delivered into the midgut via a gastroscope. To inhibit the reflux of the microbiota suspension and the gastric acid secretion, patients received intramuscular metoclopramide (10 mg) and an intravenous proton pump inhibitor at least 1 h before FMT. For patients with a high risk of anesthesia, a nasojejunal tube was inserted into the jejunum using the guidance of X-ray fluoroscopy to deliver the transplant. Generally, 5-ASA was used as maintenance therapy outside the hospital during the follow-up period.

### 2.4. Definition of Outcomes and Safety Assessment

Clinical efficacy was evaluated by partial Mayo Scores 3 months after FMT [5]. Clinical response was defined as a partial Mayo Score decrease ≥3 and ≥30% from baseline, in addition to a decrease ≥1 in the rectal bleeding sub-score or a final rectal bleeding sub-score ≤1. Clinical remission was defined as a partial Mayo Score ≤1. Adverse events (AEs) during FMT and throughout the follow-up period were evaluated. Only FMT-related AEs are reported in this study.

### 2.5. Sample Collection and Non-Targeted Metabolomics Processing

#### 2.5.1. Sample Collection

Venous blood samples were collected after 8 h without food or water consumption, both before FMT and within 7 days post-FMT. Samples were then centrifuged at 1575× *g* (3000 RPM, TDZ5-WS, XIANGZHI, Changsha, China) for 10 min. The resulting supernatant (serum) was stored at −80 °C until metabolomics processing and analysis. All combination drugs, other than FMT, were added after all sampling was completed.

#### 2.5.2. Sample Preparation

Serum samples (100 μL) were extracted using 300 μL of 2:1 (*v*/*v*) precooled (−20 °C) methanol/acetonitrile, then vortexed for 1 min and incubated at −20 °C for 2 h. After centrifugation at 4000× *g* for 20 min at 4 °C, 300 μL of supernatant was transferred for vacuum freeze drying. The metabolites were re-suspended in 50% methanol (150 μL) and centrifuged at 4 °C, 4000× *g* for 30 min, and the supernatants were transferred to the LC–MS equipment for analysis. Quality control samples were prepared by mixing equal volumes (10 μL) of each sample to evaluate the repeatability and stability of the entire LC–MS protocol.

#### 2.5.3. LC–MS

LC–MS data were acquired using a Waters 2D UPLC system (Waters, Milton, MA, USA), coupled to a Q-Exactive mass spectrometer (Thermo Fisher Scientific, Wilmington, MA, USA) at a resolution of 70,000. Chromatographic separation was performed on a Waters ACQUITY UPLC BEH C18 column (1.7 μm, 2.1 mm × 100 mm, Waters, Milton, MA, USA), with a maintained column temperature of 45 °C. Sample analysis was conducted in both positive and negative ion modes with the full scan range at 70–1050 *m*/*z*, an aux gas heater temperature of 350 °C, and a capillary temperature of 320 °C. The positive ion mode mobile phase consisted of 0.1% formic acid (A) and 100% acetonitrile (B). The negative ion mode mobile phase consisted of 10 mM ammonium formate (A) and acetonitrile (B). The flow rate was 0.35 mL/min, and the injection volume was 5 μL. The following gradient was used: 0~1 min, 2% B; 1~9 min, 2~98% B; 9~12 min, 98% B; 12~12.1 min, 98% B~2% B; and 12.1~15 min, 2% B. Other mass spectrometric settings were a sheath gas flow rate of 40, an aux gas flow rate of 10, spray voltages of 3.8 kV/−3.2 kV, an automatic gain control (AGC) target for MS acquisitions of 3 × 10^6^, and a maximum ion injection time (IT) of 100 ms. The top three precursors were selected for subsequent MSMS fragmentation with a maximum ion IT of 50 ms, a resolution of 17,500, and an AGC of 1 × 10^5^. The stepped normalized collision energy was set to 20, 40, and 60 eV.

#### 2.5.4. Metabolomic Data Analysis

Raw data were processed using the Compound Discoverer 3.0 (Thermo Fisher Scientific, Wilmington, MA, USA) to extract molecular weight, retention time, and peak area. The quality of the data was evaluated by the repeatability of tests on quality control samples. We excluded metabolites with coefficients of variation < 30%. We analyzed 1557 metabolites (positive ion mode) and 591 metabolites (negative ion mode). Metabolites were identified using a combination of several databases: BGI Library (The Beijing Genomics Institute), mzCloud, and ChemSpider (Human Metabolome Database, Kyoto Encyclopedia of Genes and Genomes, and LIPID MAPS). Metabolomic data normalization, data transformation, data scaling, and other procedures were performed using MetaboAnalyst (http://www.metaboanalyst.ca/, accessed on 22 October 2021) [15].

To screen the differential metabolites between groups, we used Welch’s *t*-test, fold change (FC) analysis, and a volcano plot for univariate analyses and orthogonal partial least squares–discriminant analysis (OPLS–DA) for multivariate statistical analyses (the threshold of variable importance of projection > 1 and *p* < 0.05). Venn diagrams displayed the intersection of differential metabolites between pairwise groups. Metabolic pathway analysis was performed using MetaboAnalyst (http://www.metaboanalyst.ca/, accessed on 22 October 2021) to reveal differential metabolites related to biological functions.

### 2.6. Statistical Analysis

All analyses were carried out using IBM SPSS Statistics 23.0.0 (SPSS Inc., Chicago, IL, USA), R version 4.0.0 (R Development Core Team, Vienna, Austria), and Python 3.7.7. Continuous data were presented as the median with the interquartile range (IQR). Categorical data were described as numbers (percentages). We compared groups using a Mann–Whitney U test for skewed continuous variables and a chi-square test or Fisher’s exact test for categorical variables. A *p*-value less than 0.05 was considered significant.

Machine learning using the “caret” package was performed to examine the complex relationships among metabolites and thus predict patient outcomes as accurately as possible. Training and testing datasets were derived by splitting the data randomly into 70% and 30% subsets, respectively. The Boruta method [16] was used in the feature selection in the training dataset, and then random forest (RF) algorithms were used to develop a classification model. We performed ten-fold cross-validation. The area under the curve (AUC) of the receiver operating characteristic curve, accuracy, sensitivity, and specificity were used to evaluate the performance of the RF classifier.

## 3. Results

### 3.1. Clinical Characteristics and Outcomes of the Participants

This study included 55 UC patients, 11 of whom were excluded for reasons described in Figure 1. Therefore, 44 patients with active UC were included in the analyses. The baseline clinical characteristics of patients are summarized in Table 1. All participants experienced an onset of UC in young adulthood (median 29.0 years, IQR 24.0–37.8). The median disease duration was 6.8 years (IQR 4.0–10.8). All patients had extensive UC: 81.8% (36/44) had pancolitis, and 18.2% (8/44) had left-sided colitis. Most participants had moderate-to-severe disease severity (95.5%, 42/44). The median Mayo Score before FMT was 10.5 (IQR 8.0–12.0), and the median endoscopic score before FMT was 3.0 (IQR 2.0–3.0). Nearly all patients had medically refractory UC (95.5%, 42/44) and had tried a variety of treatments, including 5-ASA (88.6%, 39/44), corticosteroid (86.4%, 38/44), immunosuppressant (22.7%, 10/44), and antitumor necrosis factor agents (9.1%, 4/44) before FMT, with poor response. All patients underwent FMTs through the midgut (via gastroscopy or a nasojejunal tube).

Patients with no improvement or with exacerbations in diarrhea, hematochezia, and UC-related laboratory indicators (e.g., *C*-reactive protein, white blood cell count, hemoglobin) after single FMT (Step 1) were considered for the “step-up FMT strategy” (Figure 1) described in our previous study [17]. Participants underwent a second FMT within 1 week if the response to the first transplant was inadequate (Step 2). A course of steroids, either orally or by intravenous injection, was given to those who did not respond to the second FMT. Steroids were tapered off after 2–4 weeks of full-dose oral therapy (0.75–1.0 mg/kg day prednisone; Step 3). In the present study, 18.2% (8/44) of patients received a second FMT, and steroids were prescribed to 11.4% of them (5/44, Table 1).

In total, 50.0% (22/44) and 29.5% (13/44) of patients achieved clinical response and clinical remission, respectively, 3 months post-FMT (Table 1 and Table S1). Among them, 77.3% (17/22), 9.1% (2/22), and 13.6% (3/22) had a clinical response to Step 1 (single FMT), Step 2 (two FMTs), and Step 3 (two FMTs combined with steroids), respectively. And, 76.9% (10/13), 15.3% (2/13), and 7.7% (1/13) of patients undergoing Step 1, Step 2, and Step 3 achieved clinical remission, respectively. Patients were divided into response and non-response groups, or remission and non-remission groups, for further analysis. As shown in Figure S1, UC-related symptoms improved gradually at 1, 2, 3, and 7 days post-FMT. Patients with a higher endoscopic score at baseline had a lower probability of achieving clinical response after FMT (*p* = 0.044). No statistical differences in other clinical characteristics were found between response and non-response groups or remission and non-remission groups (Table S1).

AEs occurred in 27.3% (12/44) of patients during the follow-up. Most AEs, including increased frequency of defecation, fever, abdominal pain, and itching of the skin, were transient and relieved without medical intervention. There was no difference in the occurrence of AEs in different clinical outcome groups (Table S1).

### 3.2. FMT Partly Improved the Perturbation of Serum Metabolome in UC Patients

**Figure 2:**
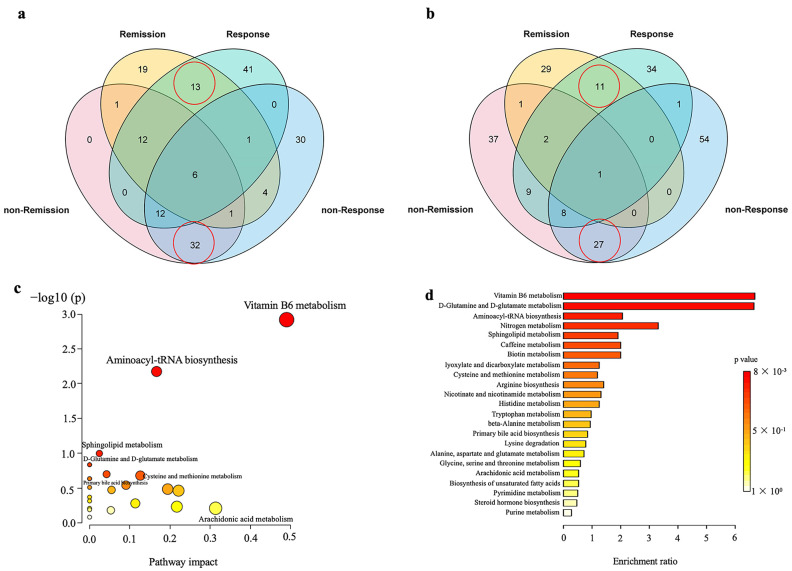
Venn diagrams illustrate the overlap and number of differential metabolites (pre- vs. post-FMT) among the various therapeutic groups in the negative mode (**a**) and positive mode (**b**). The numbers in the red circles represent the number of differential metabolites shared in the remission and response groups, non-remission and non-response groups in positive and negative ion modes, respectively. Pathway and enrichment analyses of differential metabolites (pre- vs. post-FMT) in the response group. Bubble diagram of metabolic pathway enrichment based on the differential metabolites (pre vs. post FMT) in the response group (**c**). One bubble represents one metabolic pathway. Data is plotted as significance (−log10(*p*)) versus pathway impact. The top 23 enrichment pathways are listed on the right (**d**).

One-week post-FMT, the response group samples were partially separated from baseline in the score plots of the OPLS–DA model, but no such changes were observed in other groups (Figure S2). A Venn diagram illustrated the overlap of serum metabolites among the samples and the number of metabolites in the four groups (Figure 2). There were 151 metabolites that changed significantly post-FMT in the response group (Figure 2a,b). Pathway and enrichment analyses were performed to examine the impact of FMT on the host metabolism in this group (Figure 2c,d). In the response group, the top two significantly altered pathways (*p* < 0.05), which involved eight differential metabolites, were vitamin B6 metabolism (4-pyridoxic acid, pyridoxal, 4-(phosphonooxy)-threonine), and aminoacyl-tRNA biosynthesis (L-lysine, L-serine, L-histidine, L-glutamine, and L-methionine). The eight metabolites significantly increased in the response group but not in the non-response group after FMT. Furthermore, the concentrations of baseline 4-pyridoxic acid, L-lysine, and L-serine in the response group were significantly lower than those in the non-response group, and the other five metabolites show no difference between groups at baseline (Table S2). The top two significantly enriched pathways (*p* < 0.05, Figure 2d) were vitamin B6 metabolism and D-glutamine and D-glutamate metabolism (L-glutamine).

**Figure 3:**
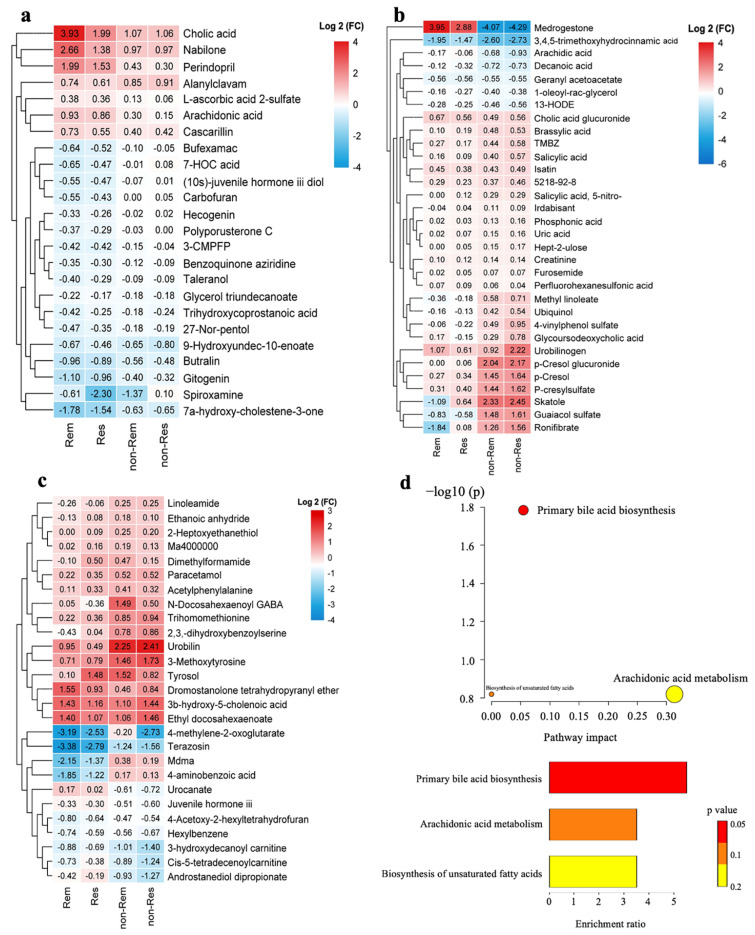
Hierarchical clustering of the differential metabolites (pre- vs. post-FMT) in the four groups (**a**–**c**). The color indicates the fold change (FC) of metabolites after FMT in this group of samples, red indicates up-regulated and blue indicates down-regulated. Differential metabolites (pre- vs. post-FMT) shared in the response and remission groups (**a**). Differential negative (**b**) and positive metabolites (**c**) shared in the non-response and non-remission groups. Pathway and enrichment analyses of differential metabolites (pre- vs. post-FMT) shared in the response and remission groups (**d**).

We found that most metabolites experienced the same trend after FMT, regardless of whether remission or response was achieved (Figure 3). In the remission and response groups, cholic acid (CA), nabilone, L-ascorbic acid-2-sulfate, arachidonic acid, and cascarillin significantly increased after FMT, and some pro-inflammatory or toxic metabolites, such as taleranol, 7α-hydroxy-4-cholesten-3-one (C4), spiroxamine, butralin, and carbofuran, show a significant decrease after intervention (Figure 3a). The primary bile acid biosynthesis pathway (CA, C4) was significantly altered and enriched in the remission and response groups (Figure 3d). In the non-remission and non-response groups, the concentrations of many toxic metabolites post-FMT were higher than the baseline (e.g., skatole, *p*-Cresol glucuronide, *p*-cresol, *p*-cresylsulfate, *N*-docosahexaenoyl gamma-aminobutyric acid, methylene dioxymethamphetamine, and 4-aminobenzoic acid), although a few protective metabolites (e.g., docosahexaenoate, tyrosol, isatin, and glycoursodeoxycholic acid) were restored by FMT (Figure 3b,c).

### 3.3. The Potential of Baseline Metabolic Profiles for Prediction of Clinical Remission of FMT

**Figure 4:**
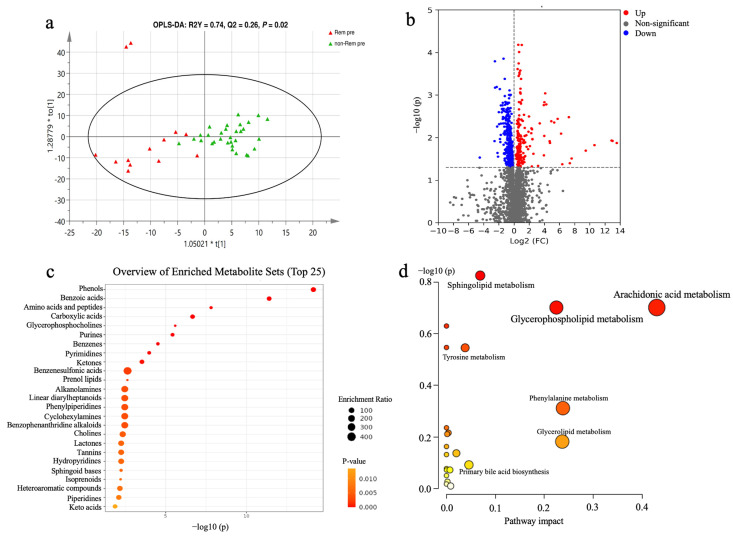
Analysis of serum metabolic profiles at baseline between the remission and non-remission groups. Comparison of the baseline serum metabolome (positive mode) of UC patients based on OPLS–DA among the remission and non-remission groups (**a**). Volcano plots of differential metabolites (positive mode) between the remission and non-remission groups; gray dots represent metabolites with no significant change, red dots represent up-regulated metabolites, and blue dots represent down-regulated metabolites (**b**). The top 25 enriched metabolite sets based on main class are listed in (**c**). Bubble diagram of metabolic pathway enrichment based on the differential metabolites between the remission and non-remission groups at baseline (**d**). OPLS–DA, orthogonal partial least squares–discriminant analysis.

We performed OPLS–DA to determine the overall differences at baseline in the remission group compared to the non-remission group and in the response group compared to the non-response group. The positive ion mode identified a clear separation between the remission and non-remission groups at baseline (Figure 4a). Indeed, Welch’s t test indicated that 430 cationic metabolites were significantly different between remission and non-remission groups at baseline (Figure 4b). The top nine significantly enriched metabolite classes were phenols, benzoic acids, amino acids (AAs) and peptides, carboxylic acids, glycerophosphocholines (GPCs), purines, benzenes, pyrimidines, and ketones (Figure 4c, *p* < 0.0001, FDR-adjusted *p*-value < 0.01). In the remission group, GPCs, glycerophospholipids, and glycerophosphoethanolamines were higher than those in the non-remission group, whereas AAs and peptide-related metabolites were lower than those in the non-remission group (Table 2). Pathway analyses of differential metabolites in the remission group included sphingolipid metabolism, glycerophospholipid metabolism, arachidonic acid metabolism, and others (Figure 4d), although with no statistical differences.

**Figure 5:**
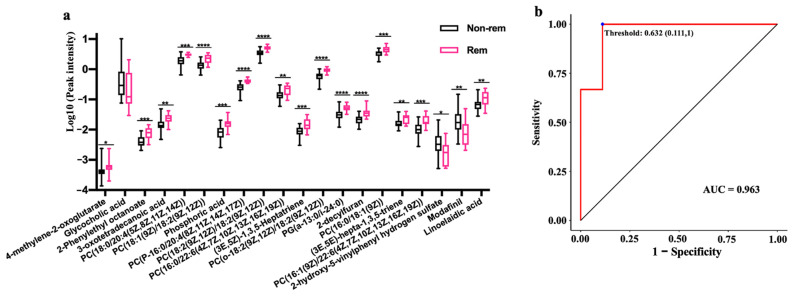
The differential metabolites at baseline between the remission and non-remission groups that were selected for the RF classifier (**a**). The RF classifier for clinical remission prediction of FMT based on serum metabolites (**b**). * 0.01 < *p* < 0.05, ** 0.001 < *p* ≤ 0.01, *** 0.001 < *p* ≤ 0.001, **** *p* ≤ 0.0001. RF, random forest.

We further explored the potential role of serum metabolites in predicting clinical remission following FMT in UC patients. Using the Boruta method [16], we selected 20 metabolites from a total of 2148 as variables for RF modeling. Nineteen of these variables were significantly different between the two groups at baseline (Figure 5a). Nine of these variables were GPCs and glycerophospholipids. The AUC, accuracy, sensitivity, and specificity of the RF classifier were 0.963, 83.3% (95% CI (51.6–97.9%)), 88.9, and 66.7%, respectively (Figure 5b).

## 4. Discussion

There is considerable interest in therapeutic microbial manipulation using FMT to treat microbiota-related diseases. In this selected population, FMT successfully induced the clinical response (50.0%, 22/44) and clinical remission (29.5%, 13/44) of UC and caused rapid improvement of symptoms within 1 week post-FMT. Participants in our study were all patients with extensive UC refractory to conventional treatments, and the uniformity of study subjects made our findings more reliable. These results were consistent with previous studies suggesting that FMT is effective for active UC [2,3,4]. The second FMT and FMTs combined with steroids contributed to an additional clinical response in 22.7% (5/22) and clinical remission in 23.1% (3/13) of participants, respectively. Though it is difficult to confirm the mechanism of the “step-up FMT strategy”, given the important role of gut microbiota in drug metabolism and immunity modulation, the effective reestablishment of gut microbiota is hypothesized to be the critical way to improve patients’ abilities to respond to other therapies [17]. FMT and immunotherapy have been reported to change the gut microbiota and reprogram the tumor microenvironment to overcome resistance to immunotherapy in patients with advanced melanoma [10].

Metabolomics refers to the analysis of patterns of small molecular metabolites in biological samples, which allows the generation of a “metabolic barcode” representing the current metabolic status of individuals under specific conditions [18,19]. Here, we profiled the changes in serum metabolites in UC patients undergoing FMT and compared the differences between various therapeutic groups. The findings of this study provide insights into the metabolic characteristics of FMT as a therapeutic approach for UC. This enriches the scientific explanations of microbiota medicine as a branch discipline in clinical medicine [20].

Metabolites involved in vitamin B6 metabolism were significantly increased in patients exhibiting a response to FMT. Vitamin B6 levels in the gut are dependent on both diet and the resident microbiota [21]. Vitamin B6 can directly scavenge reactive oxygen species [21,22], and a robust inverse relationship exists between vitamin B6 and inflammation markers [23]. It is relatively common for patients with IBD to have a vitamin B6 deficiency [24,25,26], and the prevalence of low vitamin B6 can be higher in patients with active IBD compared to patients with quiescent disease [24]. In the present study, vitamin B6 (pyridoxal) and the important metabolites involved in the synthesis and supply of pyridoxal (4-pyridoxic acid and 4-(phosphonooxy)-threonine) were significantly increased after FMT in the response group, while there was no significant increase in the non-response group. A similar response was found in an animal experiment: the amelioration of social deficits in autistic mice was associated with the increase of vitamin B6 observed after FMT [21]. As reported by Tawk Carloline et al., vitamin B6 is an important molecule for mediating host-pathogen-microbiome interactions [22]. Therefore, the restoration in vitamin B6 metabolism after successful microbiota reestablishment may promote the response observed in patients with UC. A possible mechanism is the mobilization of vitamin B6 to the sites of inflammation, where it may serve as a co-factor in pathways producing metabolites with immunomodulating effects [27].

Vitamin B6 and its interconvertible vitamers are essential co-factors involved in AA synthesis, decarboxylation, transamination, and other pathways. Consistently, we also found aminoacyl-tRNA biosynthesis-related metabolites (L-lysine, L-serine, L-histidine, L-glutamine, and L-methionine) were upregulated after FMT in the response group. AA interaction with gut microbiota has been well established in maintaining mucosal integrity and barrier function [8]. Serum AAs were frequently reported at lower concentrations in patients with IBD [1,8,28]. The supplementation of certain AAs (e.g., serine, histidine, glutamine, and methionine) has been reported to promote the alleviation of inflammation and mucosal healing by inhibiting proinflammatory cytokine production, improving intestinal antioxidant capacity and the abundance of tight junction proteins, and restoring gut microbiota [19,29,30]. Combining our results, we suspect that the restoration of AA metabolism by FMT contributes to the improvement of UC.

The main changes in serum metabolites after FMT in the remission and response groups were the increase in anti-inflammatory, antioxidant, and analgesic-related metabolites and the decrease in pro-inflammatory and toxic metabolites. The dysmetabolism of bile acids (BAs, including malabsorption and decreased conversion of primary BAs to secondary BAs) [31,32] has been reported in UC pathogenesis, during which the levels of CA, deoxycholic acid (DCA, the major secondary BAs in the gut) and secondary BAs-producing bacteria were significantly reduced [32,33]. We noted that, in responsive patients, FMT restored BA homeostasis, with increases in CA and DCA and a decrease in hydrophobic C4. Another study has also suggested that the correction of BA metabolism and bile salt hydrolase functional activity was likely the major mechanism by which FMT contributed to the cure and recurrence prevention of *Clorindiones difficile* infection [34]. The rapid improvement of diarrhea and pain after FMT can be attributed to changes in several potent molecules, such as the substantial decreases in C4 (a biomarker of BA diarrhea [31]) and 13-HODE (the endogenous agonists of transient receptor potential vanilloid-1 [35]) and the marked increase in the cannabinoid receptor 1 agonist nabilone [36]. Several toxic metabolites (e.g., skatole, *p*-cresol glucuronide, *p*-cresol, and *p*-cresylsulfate) originate in the intestine, where the gut microbiota metabolize aromatic AAs, leading to impairment of the structure and function of the intestinal epithelial barrier [37,38]. The accumulation of these toxic microbial metabolites, which are difficult to remove by dialysis, might relate to the failure of FMT in some UC patients in this study.

The prolonged inflammation and compromised integrity of the gut barrier in patients with IBD have been reported to be associated with reduced levels of phospholipids [28,39]. When comparing the metabolic profile at baseline between patients in the remission and non-remission groups, we found that patients with higher levels of GPCs were more likely to achieve clinical remission after FMT. The prominent role of GPCs in the predictive analysis provides further support that they improve FMT efficacy. Pathways of sphingolipid metabolism and glycerophospholipid metabolism were associated with positive outcomes of FMT, which were consistent with previous studies [19,40]. As a component of phosphatidylcholine (PC), GPCs may play a role in the treatment of IBD by modulating gut barrier function, remodeling gut microbiota structure and related metabolism, and reducing the inflammatory response [41]. In a randomized, placebo-controlled, multicenter trial, the highest dose of PC significantly reduced the Simple Clinical Colitis Activity Index of patients with mesalamine-refractory UC when compared to placebo, although no marked improvement in mucosal healing was observed in either group [42]. Therefore, PC supplementation before FMT may increase the clinical remission rate in patients with UC, but this possibility requires further investigation.

Finally, we identified a serum metabolite signature composed of twenty metabolites that can be used to differentiate between UC patients who may achieve clinical remission after FMT and those who may not. We used the Boruta method to select all optimally relevant features useful for efficacy prediction rather than selecting minimally optimal features. The Boruta method is the preferred variable-selection method for classification prediction modeling of datasets with many predictors due to its computational efficiency [16], making the variables used in the predictive model reliable and important.

However, some limitations existed in our study. Firstly, we could not completely account for the complex and bidirectional interactions among diet, drugs, and gut microbiota metabolism. Secondly, this was a retrospective analysis of a prospective trial with a small sample size that lacked a control group without FMT treatment. Therefore, the findings are only preliminary results that should be further validated in a large-scale and well-designed investigation. Finally, the lack of gut microbial analysis of the recipients and donors before and after FMT makes it difficult to correlate the findings directly to changes in the gut microbiota.

## 5. Conclusions

FMT effectively induced a response in patients with extensive colitis and medically refractory UC. The improvements in vitamin B6, AA, and BA metabolism in the response group suggest that effective reestablishment of gut microbiota composition had direct or indirect effects on reshaping host metabolism. Importantly, our study suggests a promising role for serum metabolites in the non-invasive prediction of FMT efficacy for treating active UC. Metabolome-informed FMT, for example, via oral PC supplementation before FMT, may provide enhanced therapeutic effects.

## Figures and Tables

**Table 1:** Clinical characteristics of the study population.

Characteristics	Results
Total number, N	44
Age, years, median (IQR)	34.5 (31.0–46.8)
Male, n (%)	25 (56.8)
Age at onset, years, median (IQR)	29.0 (24.0–37.8)
Duration of disease, years, median (IQR)	6.8 (4.0–10.8)
The Montreal classification, n (%)	
E1, proctitis	0 (0.0)
E2, left-sided colitis	8 (18.2)
E3, pancolitis	36 (81.8)
Endoscopic score before FMT, median (IQR)	3.0 (2.0–3.0)
Mayo score before FMT, median (IQR)	10.5 (8.0–12.0)
Disease severity, n (%)	
Mild	2 (4.5)
Moderate	20 (45.5)
Severe	22 (50.0)
Smoking history ^1^, n (%)	8 (18.2)
History of anal fistula surgery, n (%)	7 (15.9)
Treatment history before FMT, n (%)	
5-aminosalicylate	39 (88.6)
Corticosteroid	38 (86.4)
Immunosuppressant	10 (22.7)
Anti-TNF antibody	4 (9.1)
Step-up FMT strategy	
Step 1	36 (81.8)
Step 2	3 (6.8)
Step 3	5 (11.4)
Clinical efficacy at three months after FMT	22 (50.0)
Clinical response, n (%)	13 (29.5)
Clinical remission, n (%)	12 (27.3)
Adverse events, n (%)	

^1^ Current smoker or former smoker who had quit for less than 1 year. IQR, interquartile range; FMT, fecal microbiota transplantation; TNF, tumor necrosis factor.

**Table 2:** The representative hits of the enriched differential metabolite set between the remission and non-remission groups at baseline.

Metabolite Set	Metabolite	HMDB	Rem vs. Non-Rem	
			FC	*p* Value
Glycerophosphocholines	PC (14:0/20:4(5Z,8Z,11Z,14Z))	HMDB0007883	2.04	0.011
	PC (14:0/20:5(5Z,8Z,11Z,14Z,17Z))	HMDB0007885	3.64	0.018
	PC (16:0/18:1(9Z))	HMDB0007972	1.39	0.005
	PC (16:0/22:5(7Z,10Z,13Z,16Z,19Z))	HMDB0007990	1.41	0.018
	PC (16:0/22:6(4Z,7Z,10Z,13Z,16Z,19Z))	HMDB0007991	1.48	0.003
	PC (16:1(9Z)/22:6(4Z,7Z,10Z,13Z,16Z,19Z))	HMDB0008023	1.61	0.0002
	PC (18:0/20:4(5Z,8Z,11Z,14Z))	HMDB0008048	1.45	0.0004
	PC (18:1(9Z)/18:2(9Z,12Z))	HMDB0008105	1.65	0.0003
	PC (18:1(9Z)/22:6(4Z,7Z,10Z,13Z,16Z,19Z))	HMDB0008123	1.44	0.038
	PC (18:2(9Z,12Z)/18:2(9Z,12Z))	HMDB0008138	9.4	0.00007
	PC (18:3(9Z,12Z,15Z)/18:2(9Z,12Z))	HMDB0008204	1.85	0.0002
	PC (18:3(9Z,12Z,15Z)/18:3(9Z,12Z,15Z))	HMDB0008206	2.1	0.009
	PC (22:6(4Z,7Z,10Z,13Z,16Z,19Z)/18:0)	HMDB0008727	1.5	0.006
	PC (P-16:0/16:0)	HMDB0011206	1.46	0.028
	PC (P-16:0/18:2(9Z,12Z))	HMDB0011211	1.59	0.005
	PC (P-16:0/20:4(8Z,11Z,14Z,17Z))	HMDB0011221	1.6	0.0001
	PC (P-18:0/20:4(5Z,8Z,11Z,14Z))	HMDB0011253	1.35	0.004
	PC (P-18:0/22:6(4Z,7Z,10Z,13Z,16Z,19Z))	HMDB0011262	1.37	0.031
	PC (o-18:2(9Z,12Z)/18:2(9Z,12Z))	HMDB0013435	1.53	0.0002
Glycerophospholipids	PC (20:4(8Z,11Z,14Z,17Z)/20:5(5Z,8Z,11Z,14Z,17Z))	HMDB0008478	1.33	0.010
	PC (20:4(8Z,11Z,14Z,17Z)/22:5(7Z,10Z,13Z,16Z,19Z))	HMDB0008484	1.32	0.029
	PC (22:6(4Z,7Z,10Z,13Z,16Z,19Z)/P-16:0)	HMDB0008751	1.57	0.001
	PE (18:0/22:5(4Z,7Z,10Z,13Z,16Z))	HMDB0009010	1.91	0.009
	PE (20:4(8Z,11Z,14Z,17Z)/22:2(13Z,16Z))	HMDB0009437	1.65	0.018
	PC (P-18:1(11Z)/22:6(4Z,7Z,10Z,13Z,16Z,19Z))	HMDB0011295	1.21	0.025
	PI (16:0/16:1(9Z))	HMDB0009779	0.83	0.018
Glycerophosphoethanolamines	PE (18:0/18:1(9Z))	HMDB0008993	1.76	0.033
	PE (18:0/20:4(5Z,8Z,11Z,14Z))	HMDB0009003	2.04	0.00007
	PE (18:0/22:4(7Z,10Z,13Z,16Z))	HMDB0009009	1.91	0.002
	PE (18:0/22:6(4Z,7Z,10Z,13Z,16Z,19Z))	HMDB0009012	1.78	0.014
	PE (18:1(9Z)/18:1(9Z))	HMDB0009059	1.43	0.020
Amino acids and peptides	L-Asparagine	HMDB0000168	0.88	0.015
	Allantoic acid	HMDB0001209	0.75	0.024
	Lisinopril	HMDB0001938	0.73	0.021
	D-Ornithine	HMDB0003374	0.75	0.008
	N2-gamma-Glutamylglutamine	HMDB0011738	0.72	0.043
	*N*-Acetyl valine	HMDB0011757	0.63	0.018
	*N*-Decanoyl glycine	HMDB0013267	0.64	0.008
	Perindopril	HMDB0014928	0.27	0.0007
	Glutamyl tryptophan	HMDB0028830	0.51	0.002
	Tryptophyl-Lysine	HMDB0029088	0.7	0.011

FC, fold change.

## Data Availability

Datasets generated for this study are included in the article/supplementary material. Additional data and materials underlying this article will be shared on reasonable request to the corresponding author.

## Supplementary Materials

The following supporting information can be downloaded at: https://www.mdpi.com/article/10.3390/nu15153340/s1, Table S1: Clinical characteristics of all study populations among different groups; Table S2: The changes and importance of metabolites covered in the significant altered pathways in the response group; Figure S1: The changes in abdominal pain and daily defecation number in UC patients within 7 days after FMT; Figure S2: Comparison of serum metabolome of UC patients before and after FMT based on OPLS–DA among various therapeutic groups. OPLS–DA, orthogonal partial least squares–discriminant analysis. References [21,23,24,25,27,43,44,45,46,47,48,49,50,51,52,53,54,55,56] are cited in the supplementary materials.
