# Supplementary material for: The Underlying Changes in Serum Metabolic Profiles and Efficacy Prediction in Patients with Extensive Ulcerative Colitis Undergoing Fecal Microbiota Transplantation
Source: Nutrients. 2023 Jul 27;15(15):3340. doi: 10.3390/nu15153340 (PMC10421017; doi:10.3390/nu15153340)

Table S1 Clinical characteristics of all study populations among different groups

Items	Res (N = 22)	non-Res (N = 22)	P	Rem (N = 13)	non-Rem (N = 31)	P
Age (y), median (IQR)	32.5 (30.8 – 38.3)	42.0 (31.8 – 49.5)	0.162	32.0 (29.0 – 36.0)	36.0 (32.0 – 48.0)	0.077
Male, n (%)	11 (50.0)	14 (63.6)	0.361	5 (38.5)	20 (64.5)	0.111
The onset age (y), median (IQR)	29.0 (24.0 – 33.0)	31.0 (25.3 – 40.0)	0.371	26.0 (23.0 – 39.0)	31.0 (26.0 – 39.0)	0.091
Duration of disease (y), median (IQR)	6.3 (3.8 – 8.3)	7.0 (4.0-11.3)	0.480	8.0 (4.0 – 9.5)	6.0 (4.0 – 11.0)	0.804
The Montreal classification, n (%)			1.000			0.209
E1, proctitis	0 (0.0)	0 (0.0)		0 (0.0)	0 (0.0)	
E2, left-sided colitis	4 (18.2)	4 (18.2)		4 (30.8)	4 (12.9)	
E3, pancolitis	18 (81.8)	18 (81.8)		9 (69.2)	27 87.1)	
Endoscopic score before FMT, median (IQR)	3 (2 – 3)	3 (3 – 3)	0.044	3 (2 – 3)	3 (2 – 3)	0.628
Mayo score before FMT, median (IQR)	9.5 (7 – 11.3)	11 (9 – 12)	0.091	11 (6.5 – 11)	10 (9 – 12)	0.135
Disease severity, n (%)			0.551			0.081
Mild	2 (9.1)	0 (0.0)		2 (15.4)	0 (0.0)	
Moderate	10 (45.5)	10 (45.5)		4 (30.8)	16 (51.6)	
Severe	10 (45.5)	12 (54.5)		7 (53.8)	15 (48.4)	
Smoking history*, n (%)	3 (13.6)	5 (22.7)	0.698	2 (15.4)	6 (19.4)	1.000

History of anal fistula surgery, n (%)	5 (22.7)	2 (9.1)	0.412	2 (15.4)	5 (16.1)	1.000
Treatment history before FMT, n (%)						
5-aminosalicylate	20 (90.9)	19 (86.4)	1.000	11 (84.6)	28 (90.3)	0.623
Corticosteroid	21 (95.5)	17 (77.3)	0.185	13 (100.0)	25 (80.6)	0.157
Immunosuppressant	4 (18.2)	6 (27.3)	0.472	1 (7.7)	9 (29.0)	0.237
Anti-TNF antibody	1 (4.5)	3 (13.6)	0.607	0 (0.0)	4 (12.9)	0.302
Adverse events, n (%)	6 (27.3)	6 (27.3)	1.000	4 (30.8)	8 (25.8)	0.727

*Current smoker or former smoker who had quit for less than 1 year

Res, response; non-Res, non-response; Rem, remission; non-Rem, non-remission; IQR, interquartile range; FMT, fecal microbiota transplantation; TNF, tumor necrosis factor.

Table S2 The changes and importance of metabolites covered in the significant altered pathways in the response group

Pathway	Metabolite	Res group Post/Pre-FMT		non-Res group Post/Pre-FMT		Res vs. non-Res pre-FMT		Importance
		FC	*p* value	FC	*p* value	FC	*p* value	
Vitamin B6 metabolism	4-Pyridoxic acid	2.15	0.016	--	--	0.44	0.039	● 4-Pyridoxic acid is formed by the action of aldehyde oxidase I (an endogenous enzyme) and by microbial enzymes (pyridoxal 4-dehydrogenase)<br>● Pyridoxal is one form of vitamin B6. In humans, pyridoxal is involved in glycine and serine metabolism.<br>● There exists a robust relationship between vitamin B6 and inflammation[23]. A possible mechanism involved is mobilization
	Pyridoxal	2.6	0.014	--	--	--	--	
	4-(phosphonooxy)-threonine	2.01	0.021	--	--	--	--	

								of vitamin B6 to the sites of inflammation where it may serve as a co-factor in pathways producing metabolites with immunomodulating effects[27].<br>● Deficiencies in vitamin B6 was common in IBD, and the prevalence of low vitamin B6 was higher amongst active IBD compared to quiescent disease[24, 25].<br>● The underlying mechanism of the gut microbiota regulated the vitamin B6 levels probably by regulating the absorption and synthesis of vitamin B6 in the intestine through improving bacterial composition and gut pH[21].

<table>
<tr><td>D-Glutamine and D-glutamate metabolism</td><td>L-glutamine</td><td>1.36</td><td>0.040</td><td>--</td><td>--</td><td>--</td><td>--</td><td rowspan="2">● An important gut nutrient required by enterocytes.
● It can increase intestinal-friendly bacteria (Bacteroidetes and Actinobacteria), while decrease pernicious microbiota (Oscillospira and Treponema)[43].
● Glutamine administration was found to protect against intestinal damage and preserve gastrointestinal function by regulation several signal transduction pathways, such as NF-κB, phosphatidylinositol-3-kinase/protein kinase B, and signal transducers and activators of transcription[44-46].</td></tr>
<tr><td>aminoacyl-tRNA biosynthesis</td><td>L-glutamine</td><td>1.36</td><td>0.040</td><td>--</td><td>--</td><td>--</td><td>--</td></tr>
</table>

								● In IBD, it decreased malondialdehyde level, and increased glutathione level and superoxide dismutase activity, suggesting the activity of antioxidant, antiapoptotic, and anti-inflammation[47, 48].
	L-lysine	1.19	0.045	--	--	0.84	0.028	● Several amino acid levels, such as L-alanine, L-lysine, L-isoleucine, L-tryptophan, L-serine, were significant lower in the DSS-treated group compared with the control group [49]. ● Dietary serine supplementation improved mucin production, restored gut microbiota, protected gut epithelium, and promoted mucosal healing in DSS-treated rats[50].
	L-serine	1.28	0.013	--	--	0.88	0.033	

	L-histidine	1.24	0.026	--	--	--	--	● Histidine has been reported at lower concentrations in IBD and decreased plasma histidine level was identified as a prognostic marker for relapse in UC in six month[51, 52].<br>● Dietary histidine could ameliorate colitis by inhibition of NF-κB activation and down-regulation of proinflammatory cytokine production by macrophages[53].
	L-methionine	1.19	0.037	1.14	0.014	--	--	● L-methionine supplementation maintained the integrity and barrier function of intestinal mucosa through improving intestinal antioxidant capacity, transepithelial electrical

								resistance, and tight junction proteins abundance[54, 55].<br>● Methionine deficiency caused ETEC adhesion, reduced autophagy, and accelerated death of intestinal epithelial cells infected with ETEC[56].

FMT, fecal microbiota transplantation; FC, fold change; NF-κB, nuclear factor kappa B; IBD, inflammatory bowel disease; DSS, dextran sulfate sodium; UC, ulcerative colitis; ETEC, enterotoxigenic *Escherichia coli*.

Figure S1 The changes of abdominal pain and daily defecation number in UC patients within 7 days after FMT.

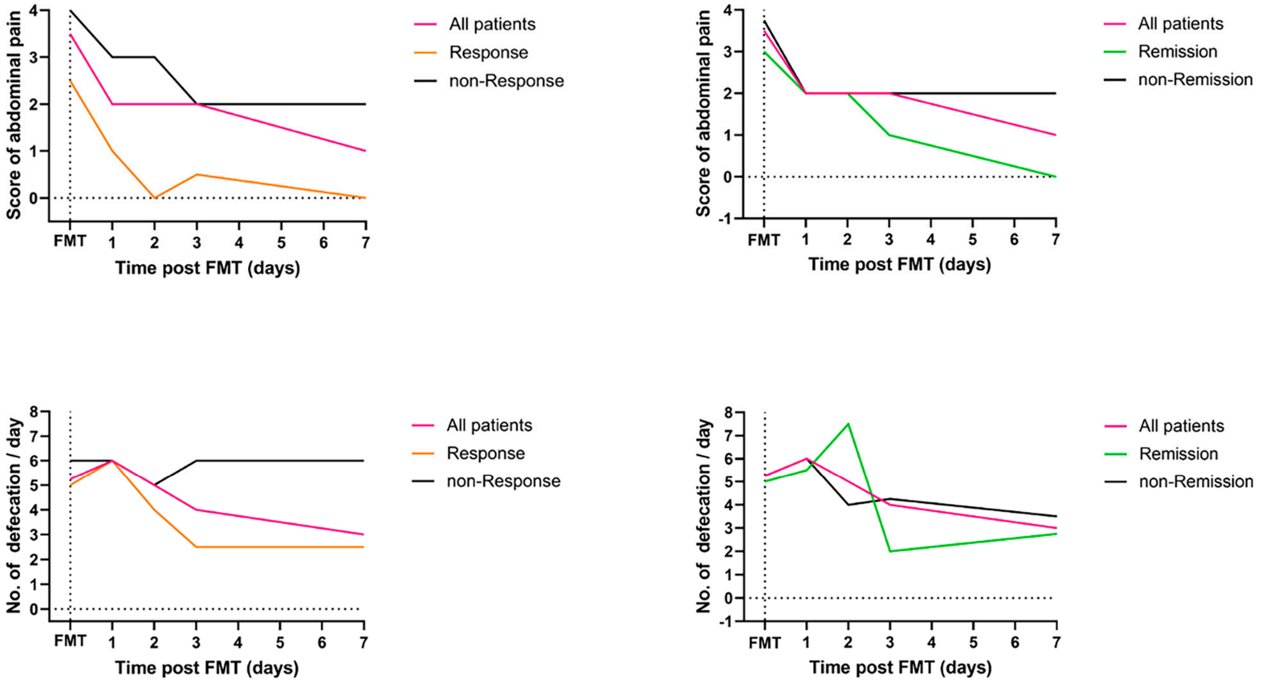

Figure S2 Comparison of serum metabolome of UC patients before and after FMT based on OPLS-DA among various therapeutic groups. OPLS-DA, orthogonal partial least squares-discriminant analysis.

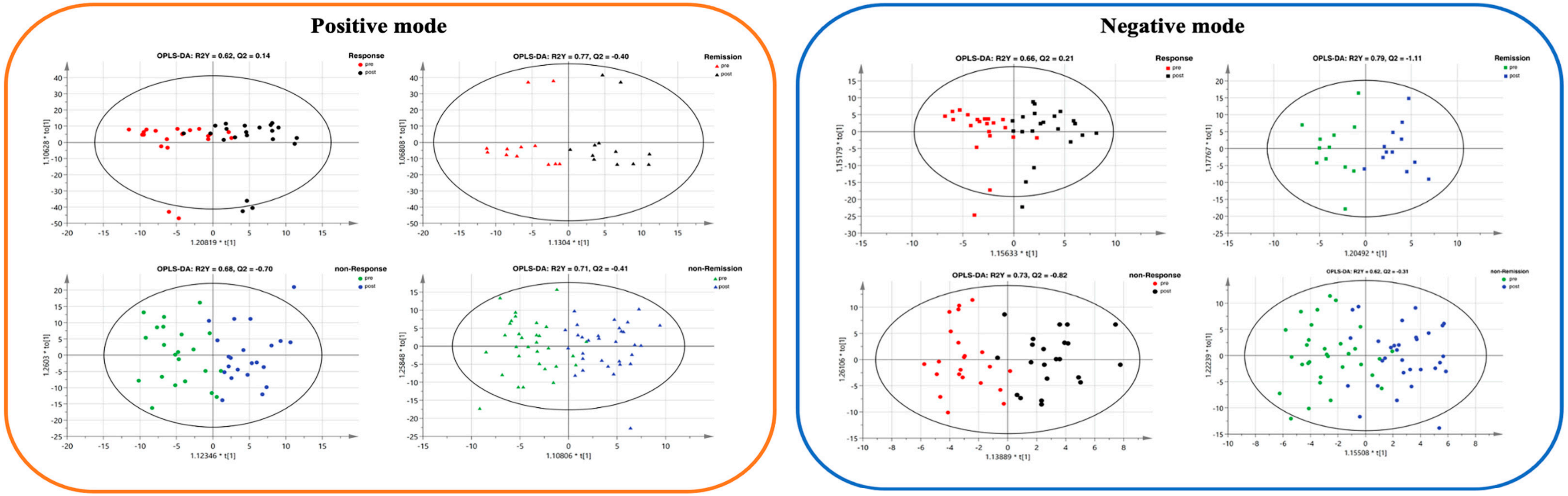

Supplement: Supplementary file 1 [file nutrients-15-03340-s001.zip › nutrients-2501825-supplementary.pdf]
